# Current treatment landscape for patients with locally recurrent inoperable or metastatic triple-negative breast cancer: a systematic literature review

**DOI:** 10.1186/s13058-019-1210-4

**Published:** 2019-12-16

**Authors:** Claire H. Li, Vassiliki Karantza, Gursel Aktan, Mallika Lala

**Affiliations:** Merck & Co., Inc., Kenilworth, NJ USA

**Keywords:** Metastatic triple-negative breast cancer, Chemotherapy, Immune checkpoint inhibitor, PARP inhibitor

## Abstract

**Background:**

Metastatic triple-negative breast cancer (mTNBC), an aggressive histological subtype, has poor prognosis. Chemotherapy remains standard of care for mTNBC, although no agent has been specifically approved for this breast cancer subtype. Instead, chemotherapies approved for metastatic breast cancer (MBC) are used for mTNBC (National Comprehensive Cancer Network Guidelines [NCCN] v1.2019). Atezolizumab in combination with nab-paclitaxel was recently approved for programmed death-ligand 1 (PD-L1)–positive locally advanced or metastatic TNBC. Published historical data were reviewed to characterize the efficacy of NCCN-recommended (v1.2016) agents as first-line (1L) and second-line or later (2L+) treatment for patients with locally recurrent inoperable or metastatic TNBC (collectively termed mTNBC herein).

**Methods:**

A systematic literature review was performed, examining clinical efficacy of therapies for mTNBC based on NCCN v1.2016 guideline recommendations. Data from 13 studies, either published retrospective mTNBC subgroup analyses based on phase III trials in MBC or phase II trials in mTNBC, were included.

**Results:**

A meta-analysis of mTNBC subgroups from three phase III trials in 1L MBC reported pooled objective response rate (ORR) of 23%, median overall survival (OS) of 17.5 months, and median progression-free survival (PFS) of 5.4 months with single-agent chemotherapy. In two subgroup analyses from a phase III study and a phase II trial (*n* = 40 each), median duration of response (DOR) to 1L chemotherapy for mTNBC was 4.4–6.6 months; therefore, responses were not durable. A meta-analysis of seven cohorts showed the pooled ORR for 2L+ chemotherapy was 11% (95% CI, 9–14%). Median DOR to 2L+ chemotherapy in mTNBC was also limited (4.2–5.9 months) per two subgroup analyses from a phase III study. No combination chemotherapy regimens recommended by NCCN v1.2016 for treatment of MBC showed superior OS to single agents.

**Conclusions:**

Chemotherapies have limited effectiveness and are associated with unfavorable toxicity profiles, highlighting a considerable unmet medical need for improved therapeutic options in mTNBC. In addition to the recently approved combination of atezolizumab and nab-paclitaxel for PD-L1–positive mTNBC, new treatments resulting in durable clinical responses, prolonged survival, and manageable safety profile would greatly benefit patients with mTNBC.

## Introduction

Breast cancer (BC) is the most common malignant neoplasm in females; an estimated 266,120 new diagnoses and 40,920 related deaths occurred in the USA in 2018 [1]. Approximately 10–20% of BCs do not express estrogen and progesterone receptors and lack amplification/overexpression of the human epidermal growth factor 2 receptor (HER2) [2–4]; therefore, they are known as triple-negative breast cancers (TNBCs) and constitute an aggressive histologic subtype. In patients with locally recurrent inoperable or metastatic disease (collectively referred to as mTNBC in this article), treatment options have primarily been chemotherapies based on recommended therapeutic approaches (National Comprehensive Cancer Network [NCCN] v1.2019 guidelines and the European School of Oncology-European Society for Medical Oncology [ESO-ESMO] 2018 guidelines) for metastatic breast cancer (MBC) [5, 6]. In particular, anthracyclines, taxanes, capecitabine, and more recently, eribulin are commonly used as monotherapy or in combination with other agents and as standard/control arms in registration trials of new/investigational agents for TNBC. Anthracyclines and taxanes are both recommended, unless contraindicated, as first-line (1L) treatments for patients who have not previously received these agents as neoadjuvant or adjuvant treatment [5, 6]. The efficacy of anthracyclines in mTNBC has been inferred from earlier studies that involved patients with MBC in which the TNBC subpopulation was not distinctly defined (mostly because of the absence of HER2 status reporting) [7]. Compared with taxanes, anthracyclines have not demonstrated overall survival (OS) benefit in mTNBC [8]. Because data on the effectiveness of anthracyclines are not available in the mTNBC population and anthracyclines and taxanes are generally considered similarly effective, anthracyclines are not discussed further in this review.

Overall prognosis for patients with mTNBC is worse than for the other BC subtypes, and more effective therapeutic options are needed. In a pooled analysis of two phase III trials in MBC, inferior outcomes were reported with 1L or later line physician choice of chemotherapy for patients with mTNBC than for the overall MBC population [9]. Chemotherapies are generally associated with unfavorable adverse events (AEs), more so in combination, that can lead to treatment discontinuation. Because combination regimens have not prolonged OS compared with monotherapies, the approach recommended by the NCCN v1.2019/ESO-ESMO 2018 guidelines [5, 6] for the treatment of MBC (including mTNBC) remains sequential use of single-agent chemotherapy. Based on recent evidence that atezolizumab plus nab-paclitaxel improves progression-free survival (PFS), this combination was recently granted accelerated approval by the US Food and Drug Administration (FDA) in patients with programmed death-ligand 1 (PD-L1)–positive (immune cell score, IC 1+) TNBC [5, 10, 11]. In general, clinical trials conducted only in patients with mTNBC are limited. No phase III trials have been conducted to specifically evaluate single agents as treatment for mTNBC in any line of therapy, and only a limited number of phase III trials have been conducted to evaluate combination therapies in the mTNBC population. The purpose of the current evidence synthesis was to systematically characterize the efficacy of commonly used chemotherapies, defined herein to be agents recommended in the NCCN v1.2016 guidelines (which were current at the time of this analysis) [12], as 1L and second-line or later (2L+) treatment for patients with mTNBC, thereby providing a summary of available historical data.

## Methods

### Systematic review

**Fig. 1:**
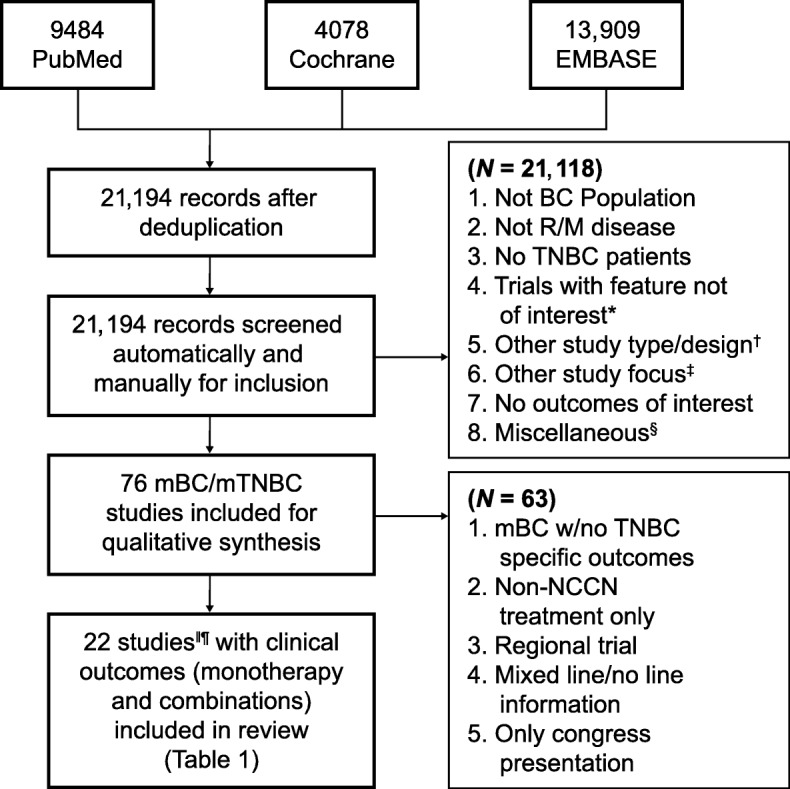
Study selection process for the systematic literature review and meta-analysis of breast cancer (*BC*). *Exclusions include not phase II, not phase III or phase II with triple-negative breast cancer (*TNBC*) focus, phase II not TNBC focus, not phase II or phase III, and not TNBC focus phase II/I. ^†^Exclusions include review articles, other study types, not recurrent/metastatic (*R/M*) of phase III/II data, and not TNBC-specific R/M. ^‡^Exclusions include non-cancer outcomes focus, only quality-of-life data, study protocol, surgical intervention, model development, and only AE data. ^§^Exclusions include other language, older report of the same study, and reference unavailable. ^‖^Results from one study (phase III trial, study 301) based on internal communication with sponsor (Eisai); not published results. ^¶^Results from Twelves et al.’s [9] and Pivot et al.’s [13] studies are both included based on the reported different treatment line outcomes

A systematic review of the literature was conducted to synthesize objective response rate (ORR), duration of response (DOR), PFS, and OS of commonly used chemotherapies as 1L or 2L+ treatment for patients with mTNBC. Commonly used chemotherapies were defined as agents recommended in the NCCN v1.2016 guidelines for the treatment of MBC (including mTNBC) as single agents or combinations thereof, including the combination of paclitaxel and bevacizumab [12]. Clinical trial results published in English between January 1, 1996, and August 21, 2016, were identified by searching the PubMed (MEDLINE), Cochrane, and Embase databases (Additional file 1: Table S1, Additional file 2: Table S2, Additional file 3: Table S3). Identified publications were then manually screened for inclusion. Reports of phase III trials in either mTNBC or MBC (with mTNBC subgroup outcomes) populations, recent (2010 and later) phase II trials in mTNBC-only populations, and retrospective or meta-analyses of mTNBC subgroups based on phase III MBC trials were included. Details of the search inclusion and exclusion criteria are presented in Fig. 1. Studies published after 21 August 2016 were evaluated separately for relevance based on recent guideline updates and were included for completeness [10, 14–19].

### Study selection

There was substantial heterogeneity in the inclusion of 1L and 2L+ populations, between and within identified studies, with many studies including mixed patient populations in terms of prior therapy and current line of treatment. Studies were first classified by line of treatment (1L, 2L+, mixed line). Only those that reported clinical efficacy outcomes in mTNBC populations in which the majority of patients (≥ 80%) were given 1L or 2L+ treatment with chemotherapy, as single-agent and in combination regimens, were included in the review. Reports of clinical trials that were conducted regionally (limited to one geographic location) in a non-White population and reports that were limited to presentation at a congress but not published were excluded from the review.

### Data analysis and meta-analysis

Clinical outcomes, including ORR and OS, were qualitatively represented by monotherapy as 1L and 2L+ therapy, as shown in Figs. 3 and 4. Meta-analyses were performed to synthesize the pooled ORRs for single-agent chemotherapy among studies of 2L+ treatment. Inverse-variance fixed-effects and random-effects meta-analyses were explored. A DerSimonian and Laird random-effects model was used to account for between-trial heterogeneity; this model assumes that the true treatment effects of the included studies follow a distribution around an overall mean [20]. The sample size, ORR, 95% confidence interval (95% CI) for each treatment and study, and pooled ORR (95% CI) are presented as forest plots, per PRISMA guidelines [21]. The ORRs were re-estimated using the all-patients-as-treated (APaT) population to ensure common definition across studies. The ORR proportions were transformed to a logit scale to calculate 95% CIs and then transformed back to proportions.

### Data sources and software

The PubMed, Cochrane, and Embase databases were searched for eligible studies/publications; Microsoft Office Excel (Redmond, WA, USA) was used to synthesize study records. As necessary, trial eligibility criteria were compared against the criteria listed on ClinicalTrials.gov. Meta-analyses of ORR were conducted in R (version 3.1.3) using the metafor package [22]. Qualitative graphical analyses of ORR, DOR, OS, and PFS across identified trials were performed using R (version 3.2.5).

## Results

A total of 21,194 references were collected from combined literature searches of PubMed, Cochrane, and Embase databases after filtering duplicate records (Fig. 1). From those references, 76 studies complied with the key inclusion criteria from qualitative synthesis. Of these 76 trials, 63 were excluded, as described in the “Results” section (Fig. 1). Finally, 21 studies that reported clinical outcomes of interest with chemotherapies for patients with mTNBC were reviewed in detail and are reported herein.

A summary of study outcomes of all included studies is given in Table 1. ORRs based on the APaT populations were calculated to facilitate comparisons across studies. ORRs were re-estimated based on the APaT population (i.e., number of responders divided by number of patients composing the APaT population for studies in which ORR was reported based on the evaluable or intention-to-treat population [ITT]). The clinical outcomes for patients with mTNBC treated with NCCN-recommended (v1.2016) agents [6, 12] as either 1L or 2L+ therapy were further separated based on whether the investigation therapy was monotherapy or combination therapy (Table 1).

**Table 1:** Study outcomes of TNBC patients treated with NCCN-recommended (v1.2016) monotherapy and combination therapy

Author	Study description	Treatment	Patient population	% 1L	% 2L	% 3L+	*N*^‡^	ORR^‡^ %	DOR, months	PFS, months	OS, months	% TNBC patients
NCCN-recommended (v1.2016) monotherapies												
Aftimos et al. [23]	Retrospective phase III sub-group analysis	Eri	2L+ MBC w/mTNBC	0	100		22	18	N/R	N/R	N/R	17
Awada et al. [24]	Phase II	Pac	1L mTNBC	100	0	0	28	28.6	N/R	3.5	13.1	100
Baselga et al. [25]	Phase II	Cis	1L–2L mTNBC	72	28	0	60	10	N/R	1.5	9.4	100
	Phase II	Cis	1L mTNBC				42	12	N/R	N/R	N/R	100
	Phase II	Cis	2L mTNBC				16	6	N/R	N/R	N/R	100
Brufsky et al. [26]	RIBBON-2	Physician’s choice chemo	2L MBC w/mTNBC	0	100	0	47	18	N/R	2.7	12.6	21
Isakoff et al. [27]	Phase II	Car/cis	1L–2L mTNBC	80	20	0	86	25.6	N/R	2.9	11	100
	Phase II	Car/cis	1L mTNBC				69	29	N/R	N/R	N/R	100
	Phase II	Car/cis	2L mTNBC				17	11.8	N/R	N/R	N/R	100
Miles et al. [28]	RIBBON-1 + AVADO + E2100 pooled subgroup	Cap/doc/pac	1L MBC w/mTNBC	100	0	0	255	23.3	N/R	5.4	17.5	26
Perez et al. [29]	BEACON	Physician’s choice chemo	3L+ MBC w/mTNBC	0	0	100	117	N/R	N/R	N/R	8.8	28
Pivot et al. [30]	Prespecified phase III subgroup	Cap	1L–3L+ MBC w/mTNBC	9	49	43	96	9	N/R	2.1	N/R	25
Sparano et al. [31]	Phase III	Cap	1L–3L+ MBC w/mTNBC	19	63	18	134	N/R	N/R	1.81	N/R	22
Study 301^†^	Phase III subgroup	Cap	1L MBC w/mTNBC				40	12	4.4	N/R	9.9	24.5
		Eri	1L MBC w/mTNBC				40	10.4	6.6	N/R	13.1	27.1
	Phase III subgroup	Cap	2L+ MBC w/mTNBC				96	~ 10	5.9	2.8	9.2	24.5
		Eri	2L+ MBC w/mTNBC				110	~ 10	4.2	3.4	15.2	27.1
Tredan et al. [32]	Phase II	Ixa	1L mTNBC	100	0	0	40	30	4.5	4.1	N/R	100
Twelves et al. [9] and Pivot et al. [13]	EMBRACE + 301 pooled subgroup	Eri	1L–3L+ MBC w/mTNBC	11	27	62	243	12	N/R	2.8	12.9	22.9
	EMBRACE + 301 pooled subgroup	Eri	2L+ MBC w/mTNBC				199	11	N/R	2.8	12.4	22.9
Twelves et al. [9] and Pivot et al. [13]	EMBRACE + 301 pooled subgroup	Physician’s choice chemo	1L–3L+ MBC w/mTNBC	13	37	50	185	10.3	N/R	2.6	8.2	23.1
	EMBRACE + 301 pooled subgroup	Physician’s choice chemo	2L+ MBC w/mTNBC				153	9	N/R	2.5	8.1	23.1
von Minckwitz et al. [33]	TANIA	Physician’s choice chemo	2L MBC w/mTNBC	0	100	0	57	N/R	N/R	2.1	N/R	23
NCCN-v1.2016-recommended combination therapies												
Brodowicz et al. [34]^§^	TURANDOT	Bev+pac	1L MBC w/mTNBC	100	0	0	63	49	N/R	9	24.2	22
Dieras et al. [35]	Phase II	Bev+pac	1L–2L mTNBC	81	19	0	61	47.53	7.5	7.2	17.4	100
	Phase II	Bev+pac	1L mTNBC				46	N/R	N/R	7.2	N/R	100
	Phase II	Bev+pac	2L mTNBC				16	N/R	N/R	7	N/R	100
Fan et al. [36]	Phase II	Doc+cap	1L mTNBC	100	0	0	26	15.4	N/R	4.8	21.5	100
Halim et al. [37]	Phase II	Car+pac	2L+ mTNBC	0	100		50	60	N/R	N/R	N/R	100
Li et al. [38]	Phase II	Cap+cis	1L–3L mTNBC	84.9	12.1	3	33	63.6	N/R	8.2	17.8	100
	Phase II	Cap+cis	1L mTNBC				28	64.3	N/R	8.2	19.6	100
	Phase II	Cap+cis	2L–3L mTNBC				5	60	N/R	5.1	16.5	100
Liao et al. [39]	Phase II	Doc+cap	1L mTNBC	100	0	0	27	14.8	N/R	4.9	21.5	100
Liao et al. [39]	Phase II	Vin+cap	1L mTNBC	100	0	0	18	27.8	N/R	5.2	18.2	100
Miles et al. [28]* (many combinations with high ORR—OS is still not much higher)	Pooled phase III (E2100, AVADO, RIBBON-1)	Bev+(cap/doc/pac/nab-pac/(dox/epi/CP/ FU))	1L MBC w/mTNBC	100	0	0	360	42.3	N/R	8.1	18.9	25
O’Shaughnessy et al. [40]	Phase III	Gem+car	1L–3L mTNBC	58	42		244	32	N/R	4.1	11.1	100
	Phase III	Gem+car	1L mTNBC				149	N/R	N/R	4.6	13.9	100
	Phase III	Gem+car	2L–3L mTNBC				109	N/R	N/R	2.9	8.1	100
Pivot et al. [30]	Prespecified phase III subgroup	Ixa+cap	1L–3L+ MBC w/mTNBC	7	48	45	91	27	N/R	4.1	N/R	24.3
Rugo et al. [41]	Phase III	Bev+nab-pac	1L MBC w/mTNBC	100	0	0	65	N/R	N/R	7.4	N/R	24
Rugo et al. [41]	Phase III	Bev+ixa	1L MBC w/mTNBC	100	0	0	63	N/R	N/R	5.6	N/R	26
Rugo et al. [41]	Phase III	Bev+pac	1L MBC w/mTNBC	100	0	0	73	N/R	N/R	6.5	N/R	26
Sparano et al. [31]	Phase III	Ixa+cap	1L–3L+ MBC w/mTNBC	20	61	19	122	N/R	N/R	4.2	N/R	20

*1L* first-line, *2L* second-line, *3L* third-line, *APaT* all patients as treated, *Bev* bevacizumab, *Cap* capecitabine, *Car* carboplatin, *chemo* chemotherapy, *Cis* cisplatin, *CP* cyclophosphamide, *Doc* docetaxel, *Dox* doxorubicin, *Epi* epirubicin, *Eri* eribulin, *FU* fluorouracil, *Gem* gemcitabine, *Ixa* ixabepilone, *MBC* metastatic breast cancer, *mTNBC* metastatic triple-negative breast cancer, *N/R* not reported, *ORR* objective response rate, *Pac* paclitaxel, *TNBC* triple-negative breast cancer, *Vin* vinorelbine

*Paclitaxel in E2100, docetaxel in AVADO, capecitabine in one cohort of RIBBON-1, and either a single-agent taxane or an anthracycline-based combination in the second cohort of RIBBON-1. Of the total *n* = 255 in the meta-analysis, *n* = 46 belong to the taxane/anthracycline cohort of RIBBON-1; the number (< 46) of this subset of patients receiving anthracycline combination is unknown

^†^Based on internal communication with trial sponsor (Eisai); not published results

^‡^*n* and ORR based on APaT population

^§^2L+ MBC with mTNBC outcomes are available in a separate study from Pivot et al. [13]

### Description of the study outcomes with NCCN-recommended (v1.2016) agents

#### Monotherapy

No published data from randomized controlled phase III trials with single-agent chemotherapy as 1L or later lines of treatment for mTNBC were found. Thirteen published reports (disregarding congress presentations) of retrospective subgroup analyses in patients with mTNBC based on phase III trials in MBC or phase II trials in mTNBC with limited sample size were identified, considering all lines of treatment. Of these, six studies reported clinical efficacy outcomes in the 1L mTNBC patient population, as summarized in Table 1 [24, 25, 27, 28, 32]. Treatments included capecitabine, taxanes (docetaxel, paclitaxel), eribulin, ixabepilone, or platinum (carboplatin, cisplatin). Furthermore, nine studies, also summarized in Table 1, reported clinical efficacy outcomes in the 2L+ mTNBC patient population; treatments included capecitabine, carboplatin, cisplatin, or eribulin [9, 13, 23, 25–27, 30, 31, 33].

##### First-line

Among the six studies on 1L treatment, five had published outcomes [24, 25, 27, 28, 32]. For one study (phase III trial, study 301), clinical outcomes for the mTNBC subgroup were available via internal communication. Notably, a meta-analysis of the mTNBC subgroups from three phase III trials in 1L MBC [28] reported a pooled ORR of 23% and median OS of 17.5 months. In trial 301, which compared eribulin with capecitabine for the treatment of MBC, in the mTNBC subgroup, ORR for 1L eribulin and capecitabine was 10% and 12%, respectively.

In addition, four phase II trials conducted to investigate single-agent chemotherapies for mTNBC with sample sizes of 28–69 were identified; the reported ORR ranged from 12 to 30%, and a median OS of 13.1 months was reported in only one [24] of these phase II trials. In studies that reported response duration (two subgroup analyses from a phase III study (study 301) and one phase II trial, all limited in sample size [*n* = 40 each]), the median DOR to 1L chemotherapy in mTNBC ranged from 4.4 to 6.6 months (Table 1) [32]. Qualitative analyses of the sample sizes, ORR, and OS are shown graphically in Figs. 3b and 4b.

##### Second-line or later

**Fig. 2:**
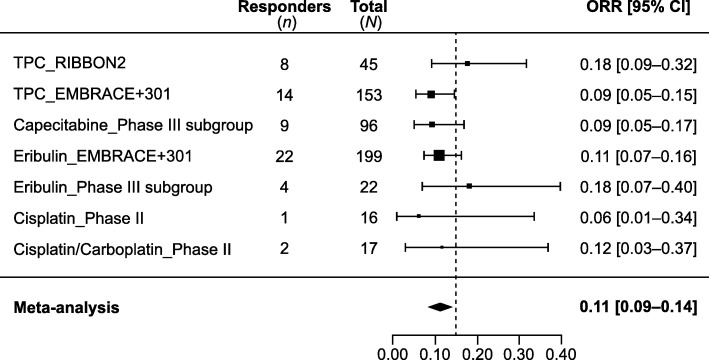
Historical objective response rate (ORR) with chemotherapy in 2L+ mTNBC. Meta-analysis of the seven cohorts from six studies reporting ORR with single-agent chemotherapy in second or later line treatment settings

**Fig. 3:**
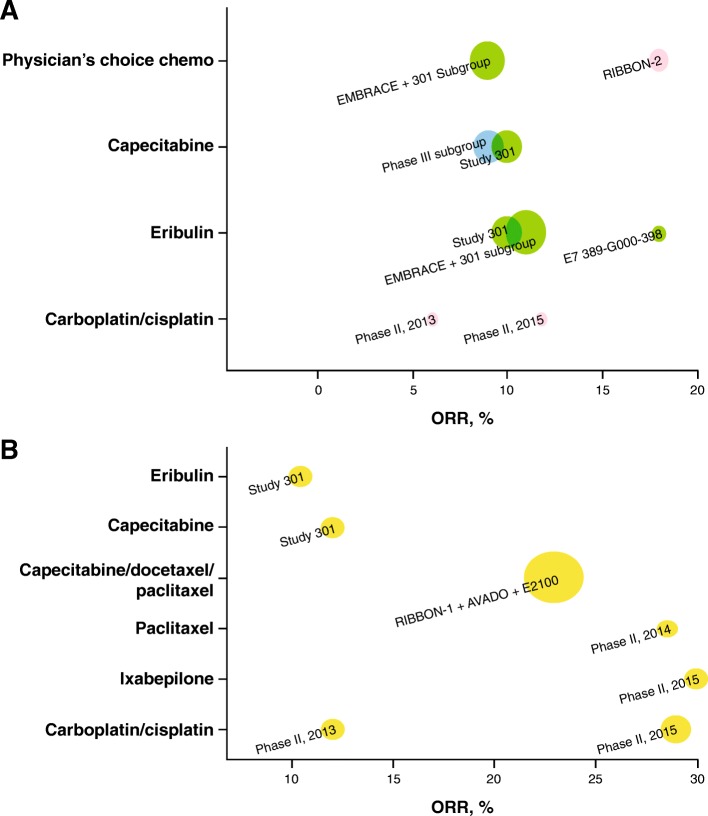
Graphical representations of objective response rates (*ORR*s) for **a** trials of NCCN-recommended (v1.2016) second-line (*2L*) plus monotherapy (including studies mixed with first-line [*1L*]), and **b** trials of NCCN-recommended (v1.2016) first-line monotherapy; the size of the bubble is proportional to the study size (all-patients-as-treated population), and the color of the bubble indicates the line of therapy. Yellow = 1L, green = 2L–3L+, pink = 2L, blue = 1L–3L+ (including studies with ≤ 15% 1L patients). Study 301 result is based on internal communication with trial sponsor (Eisai); not published results

**Fig. 4:**
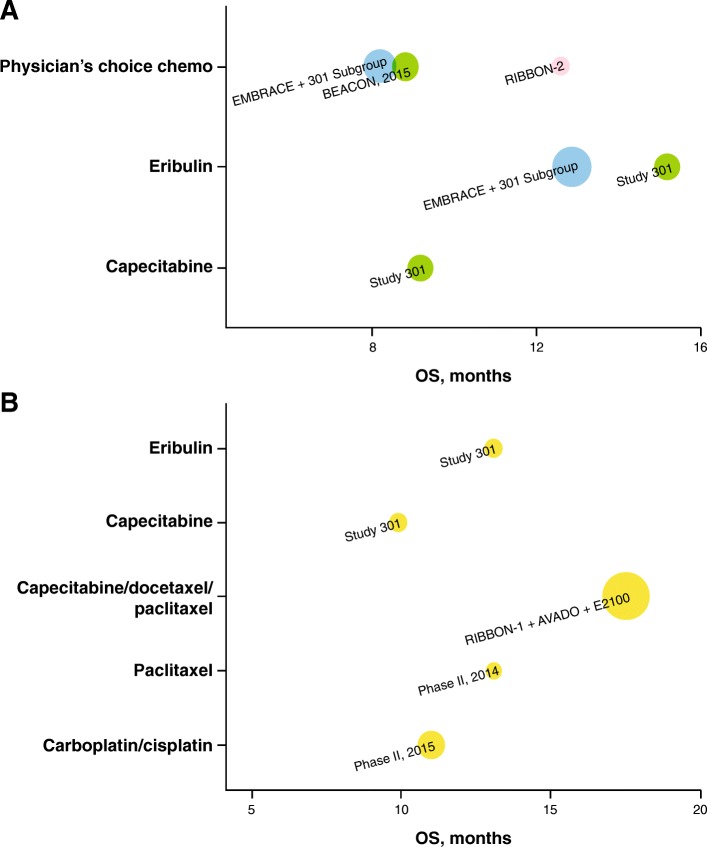
Graphical representation of overall survival (OS) for **a** trials of NCCN-recommended (v1.2016) second-line (*2L*) plus monotherapy (including studies mixed with first-line [*1L*]), and **b** trials of NCCN-recommended (v1.2016) 1L monotherapy; the size of the bubble is proportional to the study size (all-patients-as-treated population), and the color of the bubble indicates the line of therapy. Yellow = 1L, green = 2L–3L+, pink = 2L, blue = 1L–3L+ (including studies with ≤ 15% 1L patients). Study 301 result is based on internal communication with trial sponsor (Eisai); not published results. OS from phase II 2015 study is based on a total of 86 patients, including 80% 1L and 20% 2L+ patients

Among the nine studies on 2L+ treatment, seven phase III studies in MBC reported clinical efficacy outcomes for mTNBC subgroups [9, 13, 23, 26, 30, 31, 33]. In these studies, ORR ranged from 9 to 18%; median OS, from 8.1 to 15.2 months. The median DOR to 2L+ chemotherapy in mTNBC was only available from two subgroup analyses of a phase III study (study 301) and ranged from 4.2 to 5.9 months. Two additional phase II studies reported ORR of 6% and 11.8% with platinum (cisplatin/carboplatin) [25, 27]. A meta-analysis of ORR reported for seven cohorts from six of these studies (mTNBC subgroup analyses from five phase III trials in MBC and two phase II trials in mTNBC) resulted in a pooled ORR of 11% (95% CI, 9–14%) for chemotherapy in Fig. 2. Qualitative analyses of the sample sizes, ORR, and OS are shown graphically in Figs. 3a and 4a.

#### Combination therapy

Eleven published clinical studies reported efficacy outcomes in patients with mTNBC treated with NCCN-recommended (v1.2016) combination regimens [6, 12], either as chemotherapy-only regimens or in combination with bevacizumab (Table 1) [28, 30, 31, 34–41]. Only one phase III trial conducted specifically in the mTNBC population was identified, which evaluated the combination of gemcitabine, carboplatin, and iniparib/placebo as 1L–third-line (3L) treatment [40]. The overall reported ORR was 32%, and median OS was 11.1 months. In the 1L setting (*n* = 149), median PFS and OS were 4.6 and 13.9 months, respectively, whereas in the 2L+ setting (*n* = 109), median PFS and OS were 2.9 and 8.1 months, respectively.

##### First-line

In addition to monotherapy, as described previously herein, the meta-analysis of the mTNBC subgroups from three phase III trials in 1L MBC [28] also reported pooled outcomes for chemotherapy and bevacizumab combinations, including the NCCN-recommended (v1.2016) paclitaxel + bevacizumab regimen. In these studies, ORR was 42%; median OS, 18.9 months. Furthermore, two phase III trials in MBC included 20–25% patients with mTNBC and reported outcomes in their mTNBC subgroups [28, 41] In addition, four phase II trials were also identified that investigated combination regimens in mTNBC [35, 36, 38, 39]. Wide ranges of ORR (14.8–64.3%), median PFS (4.8–8.2 months), and median OS (16.5–21.5 months) were reported across these studies, in which trial designs varied and sample sizes were small (18–46 patients).

##### Second-line or later

Two phase III trials in patients with MBC reported outcomes for mTNBC subgroups treated with ixabepilone + capecitabine [30, 31]: median OS, 4.1–4.2 months, and ORR, 27% (reported in one study). An additional phase II study conducted specifically in the 2L+ mTNBC population was identified [37], which reported an ORR of 60% (*n* = 50) with the paclitaxel + carboplatin combination.

## Discussion

The current standard of care for management of mTNBC is chemotherapy, although no chemotherapy agent is specifically approved for TNBC. Instead, chemotherapies approved for MBC (all subtypes) are also used for the treatment of mTNBC (NCCN v1.2019 guidelines and ESO-ESMO guidelines 2018) [5, 6]. With the advent of immunotherapies, atezolizumab in combination with nab-paclitaxel was recently approved for PD-L1–positive locally advanced or metastatic TNBC [11]. In general, the number of clinical trials conducted only in patients with mTNBC is limited. Considering NCCN-recommended (v1.2016) treatments, there were no published phase III trials to specifically evaluate single-agent chemotherapy in mTNBC in any line of treatment and only one phase III trial that evaluated combination chemotherapy in mTNBC [40]. The most commonly used treatments were taxanes, capecitabine, and, more recently, eribulin. These agents were also used as standard/control arms in registration trials of new/investigational agents for mTNBC. The current systematic literature review was performed to determine effectiveness of treatments recommended for MBC in the NCCN v1.2016 guidelines, when used either as 1L or 2L+ therapy for mTNBC [6, 12].

The wide range of ORRs (6–29% with single agents; 14.8–64.3% with combination regimens) to NCCN-recommended (v1.2016) therapies used as 1L and 2L+ treatments for mTNBC highlights a need for more precise determination of the efficacy of these therapies to inform clinical practice. The data reviewed here suggest that the variability in ORRs was not fully attributed to differences in the effectiveness of available therapies. Small study size and heterogeneity in the characteristics of the enrolled patients (reflective of real-world clinical settings) were also significant factors. Moreover, the observed responses were generally not durable and did not necessarily translate to survival benefit. A key focus of this review was to summarize clinical outcomes taking into consideration the heterogeneity among studies caused by mixed-line patient populations and different therapeutic approaches. Historical studies identified via a systematic literature search were categorized based on the patient population being closer to 1L or later line of treatment and the regimen being monotherapy or combination.

No published results of randomized controlled phase III trials in mTNBC in 1L or later lines of treatment were found for single-agent chemotherapy. Published reports of either retrospective mTNBC subgroup analyses based on phase III trials in MBC or phase II trials in mTNBC with limited sample size were identified. These formed the evidence base in this review of historical data. A notable meta-analysis of the mTNBC subgroups from three phase III trials in 1L MBC [28] reported a pooled ORR of 23% and a median OS of 17.5 months with chemotherapy. Among available historical data, this study is regarded as the most relevant to efficacy outcomes from available 1L treatments. The recent TNT trial also reported similar clinical outcomes (31–34% ORRs and median OS of 12 months) in 2L+ mTNBC subgroups treated with carboplatin or docetaxel [19].

Although achieving clinical response is important, long-term clinical benefit of a treatment is linked with durability of the response. In two subgroup analyses from a phase III study (study 301) and one phase II trial [32], all limited in sample size (*n* = 40 each), that reported response duration, median DOR to 1L chemotherapy for mTNBC ranged from 4.4 to 6.6 months (Table 1), indicating that the responses were not durable. Considering later lines (2L+) of treatment, the efficacy of chemotherapies was lower than in the 1L setting. Based on a meta-analysis of seven cohorts, the pooled ORR for chemotherapy was 11% (95% CI, 9–14%) [13, 23, 25–27, 30]. Median DOR to 2L+ chemotherapy in mTNBC was also limited, ranging from 4.2 to 5.9 months, based on two subgroup analyses from a phase III study.

NCCN-recommended (v1.2016) combination regimens (including paclitaxel + bevacizumab) have not been proven superior to single-agent chemotherapy in terms of OS [5]. Only one global phase III trial of a combination regimen was found. This trial evaluated the gemcitabine, carboplatin, and iniparib/placebo combination as 1L–3L treatment for mTNBC [40]. Although the ORR to gemcitabine + carboplatin (32%) exceeded clinical response rates seen with monotherapy, the combination did not prolong OS (median OS, 11.1 months in 1L–3L) but was instead accompanied by higher toxicity (86% of patients had AEs of grade 3 or higher toxicities, and 10% discontinued treatment because of AEs).

In addition, the meta-analysis of the mTNBC subgroups from three phase III trials in 1L MBC [28] also reported pooled outcomes for chemotherapy and bevacizumab combinations with an ORR of 42%, which is higher than that for monotherapy, but a median OS (18.9 months) similar to that with monotherapy. The meta-analysis included patients treated with bevacizumab in combination with several chemotherapies, among which only the bevacizumab + paclitaxel combination is recommended by the NCCN v1.2016 guidelines. For 2L+ treatment of patients with mTNBC, although the ORR for combination therapies was superior to that of monotherapy, survival (median OS, 4.1–4.2 months) was poor. The current NCCN v1.2019 guidelines continue to state that the recommended approach to treatment of mTNBC remains sequential use of single-agent chemotherapy, except in patients with PD-L1–positive mTNBC, for whom atezolizumab plus nab-paclitaxel may be considered [5].

Not only do commonly used chemotherapies for MBC result in short-lived responses in patients with mTNBC, but they are also associated with toxicity, such as myelosuppression and neuropathy, which can compromise quality of life and lead to early treatment discontinuation. A pooled analysis of two phase III trials in patients with MBC (including mTNBC) receiving either single-agent physician’s choice chemotherapy (~ 70% had capecitabine) or eribulin as 2L+ treatment reported inferior outcomes with 1L or later-line chemotherapy for mTNBC than with overall MBC (ORR, 10.3% vs 16.4%; OS, 8.2 vs 12.8 months; PFS, 2.6 vs 3.4 months for chemotherapy of physician’s choice and ORR, 12.0% vs 14.9%; OS, 12.9 vs 15.2 months; PFS, 2.8 vs 4.0 months for eribulin) [9]. The study also reported that 47% and 66% of patients, respectively, for physician choice chemotherapy and eribulin, had treatment-emergent AEs of grades 3 to 4 toxicity, with neutropenia and leukopenia being the most prominent, whereas discontinuations because of treatment-emergent AEs were 13.6% and 11.3%, respectively [9]. The RIBBON-1 phase III trial [42] in patients with MBC (including mTNBC) treated in the 1L setting reported that 22% of participants in the capecitabine cohort and 38% in the taxane cohort had AEs of grade 3–5 toxicity, with sensory neuropathy, neutropenia, and venous thromboembolism being the most common. The rates of discontinuations because of AEs were 11.9% and 7.8%, respectively.

Specifically in the mTNBC population, AEs and treatment discontinuations because of toxicity have been reported in phase II studies as follows: ixabepilone (1L treatment), 45% of patients had AEs of grade ≥ 3 toxicity (neutropenia and leukopenia most common) with 20% discontinuations because of AEs [32]; paclitaxel (1L treatment), 10.7% discontinuations because of AEs [24]; and platinum (carboplatin/cisplatin, 1L or 2L treatment), 11.6% discontinuations because of AEs [27]. However, caution is required when drawing conclusions regarding the therapeutic index of different agents based on grade 3 or 4 toxicities, given that in some cases these toxicities may have minimal clinical consequence (e.g., grade 3 neutropenia in the absence of infection) whereas other chronic grade 2 toxicities may be intolerable or have a substantial impact on a patient’s quality of life.

### New agents and agents in development

New treatment options for mTNBC are emerging with the advent of immune checkpoint programmed death 1 (PD-1)/PD-L1 inhibitors, antibody drug conjugates (ADCs), and other immune therapies under investigation that could become essential for the treatment of mTNBC, either as monotherapy or in combination with other agents (Table 2). Targeted therapies and other chemotherapies under investigation, mostly in phase II studies, as 1L and later lines of treatment for mTNBC are primarily single arm and often include mixed-line patient populations; hence, efficacy outcomes are challenging to interpret [24, 43–47].

**Table 2:** Study outcomes of TNBC patients treated with NCCN-recommended (v1.2019) monotherapy in trials published since 2016

Author	Study description	Treatment	Patient population	% 1L	% 2L	% 3L+	*N*	ORR %	PFS months	OS months	% TNBC patients
Tutt et al. [19]	Phase III TNT	Car	2L+ mTNBC		100		59	31.4	3.1	12.4	100
		Doc					64	34.0	4.5	12.3	100
Kim et al. [15] and Dent et al. [14]	Phase II LOTUS	Pac+Ipatasertib	1L mTNBC	100			62	NR	6.2	NR	100
		Pac	1L mTNBC	100			62	NR	4.9	NR	100
Schmid et al. [10]	Phase III IMpassion130	Atezolizumab plus nab-paclitaxel	1L mTNBC	100			451	NR	7.2	21.3	100
		nab-paclitaxel	1L mTNBC	100			451	NR	5.5	17.6	100
Robson et al. [17, 18]	Phase III OLYMPIAD	Olaparib	1L–2L mBC		100		205	NR	7.0	19.3	49.8
		Physician’s-Choice	1L–2L mBC				97	NR	4.2	17.1	49.5
Litton et al. [16]	Phase III EMBRACA	Talazoparib	2L+ locally advanced BC		100		287	62.6	8.6	NR	45.3
		Physician’s-Choice	2L+ locally advanced BC				144	27.2	5.6	NR	41.7

*1L* first-line, *2L* second-line, *BC* breast cancer, *Car* carboplatin, *Doc* docetaxel, *mTNBC* metastatic triple-negative breast cancer, *N/R* not reported, *ORR* objective response rate, *OS* overall survival, *Pac* paclitaxel, *PFS* progression-free survival, *TNBC* triple-negative breast cancer

#### Immune checkpoint inhibitors

Compared with nab-paclitaxel alone, atezolizumab in combination with nab-paclitaxel prolonged PFS in patients with mTNBC (ITT population: median PFS of 7.2 months vs 5.5 months; Table 2) in the IMpassion 130 trial. Median PFS among the subpopulation of that trial with PD-L1–positive tumors was 7.5 months in the atezolizumab group and 5.0 months in the placebo group [10]. PD-L1 positivity in that trial was determined using the Ventana PD-L1 [SP142] immunohistochemical assay (Roche Diagnostics USA) and was defined based on the percentage of PD-L1–expressing immune cells as a percentage of tumor area: IC3 (≥ 10%), IC2 (≥ 5% to < 10%), IC1 (≥ 1% and < 5%), and IC0 (< 1%). Combination atezolizumab plus nab-paclitaxel is now approved by the FDA for the treatment of PD-L1–positive (IC1+) mTNBC (with PD-L1 positivity established using an FDA-approved test) and is included in the most recent NCCN v1.2019 guidelines [5, 11]. Results of KEYNOTE-355, a phase III study of pembrolizumab in combination with one of (nab)-paclitaxel, gemcitabine, or carboplatin as 1L therapy for mTNBC, are pending.

Immune checkpoint inhibitors are also being investigated for monotherapy, and atezolizumab and pembrolizumab both have shown durable responses but in limited patient subsets. Results from the single-arm atezolizumab monotherapy trial in mTNBC were promising, with an ORR of 26% and 7% in the 1L and 2L+ settings, respectively; median DOR was 21 months (range 8+ to 26+ months) in the 1L setting, and DOR ranged from 3 to 13+ months in the 2L+ setting [48]. In KEYNOTE-086, a phase II study of pembrolizumab monotherapy for heavily pretreated mTNBC reported an overall ORR of 5% in a 2L+ subset of patients. The median DOR was 6.3 months (range, 1.2+ to 10.3+ months), with a median PFS and OS of 2 months and 8.9 months, respectively [49].

#### PARP inhibitors

When the NCCN guidelines were updated in 2018 and 2019, after this systematic review was conducted, two poly adenosine diphosphate (ADP) ribose polymerase (PARP) inhibitors, olaparib and talazoparib, were added for the treatment of germline *BRCA*-mutated HER2-negative MBC [50]. In the recent phase 3 OlympiAD trial of single-agent olaparib versus physician choice chemotherapy as 1L+ treatment for patients with germline *BRCA*-mutant and HER2-negative MBC (50% of patients with mTNBC), use of olaparib showed improvement in ORR (60% vs 29%) and median PFS (7.0 months vs 4.2 months) compared with chemotherapy [17]. Similarly, the EMBRACA trial of talazoparib versus chemotherapy as a 2L+ treatment in a similar patient population (45% mTNBC) reported that, compared with chemotherapy, talazoparib conferred a significantly higher ORR (62.6% vs 27.2%; *P* < 0.001) and significantly longer median PFS (8.6 months vs 5.6 months; *P* < 0.001) (Table 2) [16].

#### AKT inhibitors

Addition of AKT inhibitors to chemotherapy is also being investigated as 1L treatment for patients with mTNBC. A recent combination trial of the AKT inhibitor ipatasertib plus paclitaxel as 1L treatment for mTNBC (LOTUS trial) reported a median PFS of 6.2 months with the ipatasertib combination (vs 4.9 months with the placebo combination; *P* = 0.037; Table 2). After a follow-up of 23 months, median OS was 23.1 months with ipatasertib (vs 18.4 months with placebo plus paclitaxel) and the 1-year OS rate increased from 70 to 83% with the addition of ipatasertib; OS seemed to be independent of *PTEN* expression status [14, 15]. Furthermore, the AKT inhibitor AZD5363 (capivasertib) is being investigated in combination with paclitaxel in patients with previously untreated mTNBC (PAKT) [51]. After a median follow-up of 18.2 months, PFS and OS were both longer with capivasertib plus paclitaxel than with placebo plus paclitaxel (PFS, 5.9 months vs 4.2 months; OS, 19.1 months vs 12.6 months).

#### Antibody drug conjugates

Among ADCs, on February 5, 2016, the FDA granted breakthrough therapy designation to sacituzumab govitecan (IMMU-132) as 3L treatment for mTNBC based on the results of a phase I/II clinical trial, which demonstrated an ORR of 34%, a median PFS of 5.5 months, and a median OS of 12.7 months [52]. In the EMERGE phase II trial with the 3L+ mTNBC subpopulation treated with another ADC, glembatumumab vedotin (GV), reporting an ORR of 18% (vs 0% for the chemotherapy-treated counterparts), these figures were 40% and 0%, respectively, for patients with mTNBC overexpressing glycoprotein NMB (gpNMB) [53]. There was a suggestion of possible improvement in survival (PFS and OS) with GV compared with chemotherapy in this population of the EMERGE study (PFS: 3.5 months vs 1.5 months; OS, 10.0 months vs 5.5 months) [53]. However, a recent trial of GV versus capecitabine in a similar population of patients with gpNMB-overexpressing mTNBC (METRIC) did not meet its primary PFS objective, with no improvement in PFS with GV compared with capecitabine, and no OS benefit [54].

### Limitations

No mTNBC-specific randomized controlled trials directly comparing NCCN-recommended (v1.2016) chemotherapies for the treatment of MBC were identified in this search, allowing only indirect comparison between studies. Furthermore, no phase III trial studying single-agent chemotherapy for the treatment of mTNBC in any line of therapy was found. Given that results from only one global phase III trial to evaluate combination chemotherapy in mTNBC are available [40], retrospective (and in one case prospective [41]) subgroup analyses of the mTNBC subpopulation from larger phase III MBC trials and smaller phase II trials, including single-arm trials, were included in this evidence synthesis. Furthermore, for the meta-analysis of 2L+ chemotherapies, quantitative adjustment for differences in patient characteristics across trials was not feasible because of the paucity of such historical trials. It should also be noted that these clinical trial results are representative of a very select group of patients with mTNBC. Therefore, worse outcomes are likely in the general population of patients, many of whom would not meet the stringent eligibility criteria specified in these clinical trials (e.g., exclusion of patients with brain metastases at screening, exclusion of patients with early recurrences in first-line studies).

## Conclusions

Adequately controlled historical data on the treatment of mTNBC are limited, which may be attributed to the lack of therapies specific to mTNBC. Among the available historical data, commonly used chemotherapies have demonstrated limited durability of response, limited survival benefit, and challenging toxicity profiles, suggesting a considerable unmet medical need in mTNBC. The recent approval of the combination of nab-paclitaxel and atezolizumab for the treatment of PD-L1–positive (IC1+) mTNBC is a positive development for a subset of patients with mTNBC (41% by the Ventana PD-L1 [SP142] assay). However, therapeutic regimens that result in improved, sustainable clinical responses and longer survival, along with more manageable safety profiles, are still needed for patients with mTNBC, including those with PD-L1–negative tumors. Ongoing and future studies with immune therapies, targeted agents, and ADCs, either as monotherapy or combination treatment, can provide new opportunities for improved outcomes in patients with this difficult-to-treat BC subtype.

## Supplementary information

**Additional file 1:** **Table S1.** PubMed search queries.

**Additional file 2:** **Table S2.** Cochrane search queries.

**Additional file 3:** **Table S3.** Embase search queries.

## Data Availability

Not applicable.

## Abbreviations

1L: First-line
2L+: Second-line or higher
3L: Third-line
ADCs: Antibody drug conjugates
AE: Adverse event
APaT: All patients as treated
BC: Breast cancer
CI: Confidence interval
DOR: Duration of response
ESO-ESMO: European School of Oncology-European Society for Medical Oncology
FDA: US Food and Drug Administration
gpNMB: Glycoprotein NMB
GV: Glembatumumab vedotin
MBC: Metastatic breast cancer
mTNBC: Metastatic triple-negative breast cancer
NCCN: National Comprehensive Cancer Network
ORR: Objective response rate
OS: Overall survival
PD-1: Programmed death 1
PD-L1: Programmed death-ligand 1
PFS: Progression-free survival
TNBC: Triple-negative breast cancer
