# Supplementary material for: Current treatment landscape for patients with locally recurrent inoperable or metastatic triple-negative breast cancer: a systematic literature review
Source: Breast Cancer Res. 2019 Dec 16;21:143. doi: 10.1186/s13058-019-1210-4 (PMC6916124; doi:10.1186/s13058-019-1210-4)
Supplement: Supplementary file 1 — Additional file 1: Table S1. PubMed search queries. [file 13058_2019_1210_MOESM1_ESM.docx]

**Table S1.** PubMed search queries

| **Search No.** | **PubMed Search Query** | **#Hits** |
| --- | --- | --- |
| 1 | Breast cancer[MeSH Terms] | 234418 |
| 2 | (breast) AND (cancer* OR tumor* OR tumour* OR carcinoma* OR neoplasm* OR carcinogen* OR malignan*) | 321983 |
| 3 | #1 OR #2 | 321983 |
| 4 | metastat* OR advance* OR second* OR recurren* OR inoperab* OR disseminat* OR incur* | 2729933 |
| 5 | #3 AND #4 | 92199 |
| 6 | Drug Therapy[MeSH Terms] | 1123555 |
| 7 | “Therapy, Drug” OR “Drug Therapies” OR “Therapies, Drug” OR “Chemotherapy” OR “Chemotherapies” OR “Pharmacotherapy” OR “Pharmacotherapies” OR “Drug Therapy” OR antineoplastic* OR palliat* | 2232804 |
| 8 | “abi 007” OR “abi007” OR “abraxane” OR “albumin bound paclitaxel” OR “albumin-bound paclitaxel” OR “anzatax” OR “asotax” OR “biotax” OR “bms 181339” OR “bms181339” OR “bristaxol” OR “britaxol” OR “coroxane” OR “formoxol” OR “genexol” OR “genexol pm” OR “hunxol” OR “ifaxol” OR “infinnium” OR “intaxel” OR “mbt 0206” OR “mbt0206” OR “medixel” OR “mitotax” OR “nab paclitaxel” OR “nanoparticle albumin bound paclitaxel” OR “nsc 125973” OR “nsc125973” OR “oncogel” OR “onxol” OR “pacitaxel” OR “paclitaxel nab” OR “pacxel” OR “padexol” OR “parexel” OR “paxceed” OR “paxene” OR “paxus” OR “praxel” OR “taxocris” OR “taxol” OR “taxus (drug)” OR “taycovit” OR “yewtaxan” OR “paclitaxel” | 30600 |
| 9 | “daxotel” OR “dexotel” OR “docefrez” OR “docetaxel accord” OR “lit 976” OR “lit976” OR “n debenzoyl n tert butoxycarbonyl 10 deacetyltaxol” OR “n tert butoxycarbonyl 10 deacetyl n debenzoyltaxol” OR “nsc 628503” OR “nsc628503” OR “oncodocel” OR “rp 56976” OR “rp56976” OR “taxoter” OR “taxotere” OR “texot” OR “docetaxel” | 12070 |
| 10 | “2' deoxy 2', 2' difluorocytidine” OR “2', 2' difluorodeoxycytidine” OR “difluorodeoxycytidine” OR “gemcitabine hydrochloride” OR “gemcite” OR “gemzar” OR “ly 188011” OR “ly188011” OR “gemcitabine” | 12365 |
| 11 | “5' deoxy 5 fluoro n4 (pentyloxycarbonyl) cytidine” OR “apecitab” OR “ecansya” OR “ro 09 1978” OR “ro 09-1978” OR “ro 091978” OR “ro09 1978” OR “ro09-1978” OR “ro091978” OR “xeloda” OR “capecitabine” | 33860 |
| 12 | “5' noranhydrovinblastine” OR “anhydrovinblastine, 5' nor” OR “anx 530” OR “anx530” OR “eunades” OR “exelbine” OR “kw 2307” OR “kw2307” OR “navelbin” OR “navirel” OR “vinbine” OR “vinelbine” OR “vinorelbine” OR “vinorelbine ditartrate” OR “vinorelbine tartrate” | 3516 |
| 13 | “e 7389” OR “e7389” OR “eribulin mesilate” OR “eribulin mesylate” OR “halaven” OR “eribulin” | 282 |
| 14 | “7, 11 dihydroxy 8, 8, 10, 12, 16 pentamethyl 3 [1 methyl 2 (2 methyl 4 thiazolyl) ethenyl] 17 oxa 4 azabicyclo [14.1.0] heptadecane 5, 9 dione” OR “azaepothilone B” OR “bms 247550” OR “bms 247550 1” OR “bms 247550-1” OR “bms247550” OR “bms247550 1” OR “bms247550-1” OR “ixempra” OR “ixempra kit” OR “nsc 710428” OR “nsc710428” OR “ixabepilone” | 1427814 |
| 15 | “altuzan” OR “avastin” OR “nsc 704865” OR “nsc704865” OR “bevacizumab” | 12195 |
| 16 | herceptin OR trastuzumab | 7977 |
| 17 | “2C4” OR “monoclonal antibody 2C4” OR “omnitarg” OR “perjeta” OR “r 1273” OR “r1273” OR “rhumab 2C4” OR “pertuzumab” | 1996 |
| 18 | “4 [3 chloro 4 (3 fluorobenzyloxy) anilino] 6 [5 [2 (methylsulfonyl) ethylaminomethyl] 2 furyl] quinazoline” OR “gw 2016” OR “gw 572016” OR “gw 572016f” OR “gw2016” OR “gw572016” OR “gw572016f” OR “lapatinib ditosylate” OR “lapatinib ditosylate monohydrate” OR “lapatinib tosylate” OR “n [3 chloro 4 [(3 fluorobenzyl) oxy] phenyl] 6 [5 [[[2 (methylsulfonyl) ethyl] amino] methyl] furan 2 yl] quinazolin 4 amine bis (4 methylbenzenesulfonate)” OR “tykerb” OR “tyverb” OR “lapatinib” | 7655 |
| 19 | “40 o (2 hydroxyethyl) rapamycin” OR “affinitor” OR “afinitor” OR “afinitor disperz” OR “certican” OR “nvp rad 001” OR “nvp rad001” OR “rad 001” OR “rad 001a” OR “rad001” OR “rad001a” OR “sdz rad” OR “votubia” OR “xience” OR “xience V” OR “zortress” OR “everolimus” | 4500 |
| 20 | “10 [(3 amino 2, 3, 6 trideoxy alpha levo arabino hexopyranosyl) oxy] 8 glycoloyl 7, 8, 9, 10 tetrahydro 6, 8, 11 trihydroxy 1 methoxy 5, 12 naphthacenedione” OR “3 glycoloyl 1, 2, 3, 4, 6, 11 hexahydro 3, 5, 12 trihydroxy 10 methoxy 6, 11 dioxo 1 naphthacenyl 3 amino 2, 3, 6 trideoxy alpha levo arabino hexopyranoside” OR “4 epiadriamycin” OR “4' epiadriamycin” OR “4' epidoxorubicin” OR “4' epirubicin” OR “adriamycin, 4' epi” OR “binarin” OR “doxorubicin, 4' epi” OR “ellence” OR “epi-cell” OR “epiadriamycin” OR “epidoxo” OR “epidoxorubicin” OR “epidx” OR “epifil” OR “epilem” OR “epirubicin hydrochloride” OR “farmorrubicina rtu” OR “farmorubicin” OR “farmorubicin pfs” OR “farmorubicin rd” OR “farmorubicina” OR “farmorubicina cs” OR “farmorubicina r.d.” OR “farmorubicine” OR “imi 28” OR “nsc 256942” OR “pharmorubicin” OR “pharmorubicin pdf” OR “pharmorubicin pfs” OR “pharmorubicin r.d.f.” OR “pharmorubicin rds” OR “pharmorubicine” OR “pharmorubucin rd” OR “pidorubicin” OR “epirubicin” | 51867 |
| 21 | “14 hydroxydaunomycin” OR “14 hydroxydaunorubicin” OR “a.d.mycin” OR “adriablastin” OR “adriablastina” OR “adriablastina r.d.” OR “adriablastine” OR “adriacin” OR “adriamicina” OR “adriamicine” OR “adriamycin” OR “adriamycin hydrochloride” OR “adriamycin p.f.s.” OR “adriamycin pfs” OR “adriamycin r.d.f.” OR “adriamycin rd” OR “adriamycin rdf” OR “adriamycina” OR “adriblastin” OR “adriblastina” OR “adriblastina cs” OR “adriblastina pfs” OR “adriblastine” OR “adrim” OR “adrimedac” OR “adrubicin” OR “amminac” OR “caelix” OR “caelyx” OR “caelyx/doxil” OR “carcinocin” OR “dexorubicin” OR “dox sl” OR “doxil” OR “doxil (liposomal)” OR “doxolem” OR “doxor lyo” OR “doxorubicin hydrochloride” OR “doxorubicin meiji” OR “doxorubicin, liposomal” OR “doxorubicine” OR “doxorubin” OR “evacet” OR “farmiblastina” OR “fi 106” OR “fi106” OR “ifadox” OR “lipodox” OR “liposomal doxorubicin” OR “mcc 465” OR “mcc465” OR “myocet” OR “nsc 123127” OR “nsc123127” OR “pegylated liposomal doxorubicin” OR “polyethylene glycol-coated liposomal doxorubicin” OR “rastocin” OR “resmycin” OR “rp 25253” OR “rp25253” OR “rubex” OR “rubidox” OR “sarcodoxome” OR “tlc d 99” OR “doxorubicin” | 62114 |
| 22 | “2 [bis (2 chloroethyl) amino] tetrahydro (2h) 1, 3, 2 oxazaphosphorine 2 oxide” OR “2 [bis (beta chlorethyl) amino] 1 oxa 3 aza 2 phosphacyclohexan 2 oxid” OR “2 [bis (2 chloroethyl) amino] 1 oxa 3 aza 2 phosphacyclohexane” OR “2 [bis (2 chloroethyl) amino] (2h) 1, 3, 2 oxazaphosphorinane 2 oxide” OR “2h 1, 3, 2 oxazaphosphorine 2 [bis (2 chloroethyl) amino] tetrahydro 2 oxide” OR “alkyroxan” OR “b 518” OR “b 518 asta” OR “b518” OR “b518 asta” OR “carloxan” OR “ciclofosfamida” OR “ciclolen” OR “cicloxal” OR “clafen” OR “cyclo-cell” OR “cycloblastin” OR “cycloblastine” OR “cyclofos amide” OR “cyclofosfamid” OR “cyclofosfamide” OR “cyclophar” OR “cyclophosphamid” OR “cyclophosphamide isopac” OR “cyclophosphamides” OR “cyclophosphan” OR “cyclophosphane” OR “cyclostin” OR “cyclostin n” OR “cycloxan” OR “cyphos” OR “cytophosphan” OR “cytophosphane” OR “cytoxan” OR “cytoxan lyophilized” OR “endocyclo phosphate” OR “endoxan” OR “endoxan asta” OR “endoxan-asta” OR “endoxana” OR “endoxon-asta” OR “enduxan” OR “genoxal” OR “ledoxan” OR “ledoxina” OR “lyophilized cytoxan” OR “mitoxan” OR “n, n bis (2 chlorethyl) n o propylene phosphoric acid ester diamide” OR “n, n bis (2 chloroethyl) n' 3 (hydroxypropyl) phosphorodiamidic acid intramolecular ester” OR “n, n bis (beta chlorethyl) n' ortho trimethylenphosphorsaureesterdiamid” OR “neosan” OR “neosar” OR “noristan” OR “nsc 26271” OR “nsc 2671” OR “procytox” OR “procytoxide” OR “semdoxan” OR “sendoxan” OR “syklofosfamid” OR “cyclophosphamide” | 3062860 |
| 23 | “2, 4 dioxo 5 fluoropyrimidine” OR “5 fluoro 2, 4 pyrimidinedione” OR “5 fluoro uracil” OR “5 fluoropyrimidine 2, 4 dione” OR “5 fluorouracil” OR “5 fluoruracil” OR “5 fu” OR “accusite” OR “actino-hermal” OR “adrucil” OR “agicil” OR “carac” OR “cinkef u” OR “effluderm” OR “efudex” OR “efudix” OR “efurix” OR “eurofluor” OR “f6627” OR “fivoflu” OR “fluoro uracil” OR “fluoroblastin” OR “fluoroplex” OR “fluorouracil 5” OR “fluorouracil sodium” OR “fluoruracil” OR “fluouracil” OR “fluoxan” OR “flurablastin” OR “fluracedyl” OR “fluracil” OR “fluracilium” OR “fluril” OR “fluro uracil” OR “fluroblastin” OR “fluroblastine” OR “ifacil” OR “nsc 18913” OR “nsc 19893” OR “nsc18913” OR “nsc19893” OR “oncofu” OR “ribofluor” OR “ro 2 9757” OR “ro 2-9757” OR “ro2 9757” OR “ro2-9757” OR “tolak” OR “uflahex” OR “utoral” OR “verrumal” OR “fluorouracil” | 7811021 |
| 24 | #6 OR #7 OR #8 OR #9 OR #10 OR #11 OR #12 OR #13 OR #14 OR #15 OR #16 OR #17 OR #18 OR #19 OR #20 OR #21 OR #22 OR #23 | 11533014 |
| 25 | #5 AND #24 | 62201 |
| 26 | Clinical Trial/ | 966255 |
| 27 | Clinical Trials/ | 1040880 |
| 28 | Clinical Trials as Topic/ | 295404 |
| 29 | Clinical trial[Publication Type] | 721277 |
| 30 | (phase) AND (1 OR 2 OR 3 OR 4 OR I OR II OR III OR IV) | 671171 |
| 31 | #26 OR #27 OR #28 OR #29 OR #30 | 1634268 |
| 32 | #25 AND #31 | 15705 |
| 33 | case report[Title/Abstract] | 233648 |
| 34 | review[Publication Type] | 2073271 |
| 35 | letter[Publication Type] | 904983 |
| 36 | Historical Article/ | 363047 |
| 37 | systematic review[Title/Abstract] | 70667 |
| 38 | clinical review[Title/Abstract] | 3711 |
| 39 | comment[Publication Type] | 649609 |
| 40 | case reports[Publication Type] | 1761816 |
| 41 | editorial[Publication Type] | 394887 |
| 42 | Historical Article[Publication Type] | 363035 |
| 43 | Interview[Publication Type] | 26516 |
| 44 | Letter[Publication Type] | 904983 |
| 45 | #33 OR #34 OR #35 OR #36 OR #37 OR #38 OR #39 OR #40 OR #41 OR #42 OR #43 OR #44 | 5376224 |
| 46 | #32 NOT #45 | 11508 |
| 47 | “1996”[Date - Publication] : “2016”[Date - Publication] | 14289217 |
| 48 | English[Language] | 21225712 |
| **49** | **#46 AND #47 AND #48** | **8554** |
