# Supplementary material for: Current treatment landscape for patients with locally recurrent inoperable or metastatic triple-negative breast cancer: a systematic literature review
Source: Breast Cancer Res. 2019 Dec 16;21:143. doi: 10.1186/s13058-019-1210-4 (PMC6916124; doi:10.1186/s13058-019-1210-4)
Supplement: Supplementary file 3 — Additional file 3: Table S3. Embase search queries. [file 13058_2019_1210_MOESM3_ESM.docx]

**Table S3.** Embase search queries

| **S. No.** | **EMBASE Search Query** |
| --- | --- |
| 1 | breast cancer'/exp |
| 2 | (breast) Near/5 (cancer* or tumor* or tumour* or carcinoma* or neoplasm* or carcinogen* or malignan*) |
| 3 | #1 OR #2 |
| 4 | (metastat* OR advance* OR second* OR recurren* OR inoperab* OR disseminat* OR incur*) NEAR/6 (breast) |
| 5 | (metastat* OR advance* OR second* OR recurren* OR inoperab* OR disseminat* OR incur*) NEAR/6 (cancer* or tumor* or tumour* or carcinoma* or neoplasm* or carcinogen* or malignan*) |
| 6 | #4 OR #5 |
| 7 | #3 AND #6 |
| 8 | drug therapy'/exp |
| 9 | chemoth* OR ('drug' AND thera*) OR pharmacoth* OR antineoplastic* OR palliat* |
| 10 | (Paclitaxel OR Docetaxel OR Gemcitabine OR Capecitabine OR Vinorelbine OR Eribulin OR Ixabepilone OR Bevacizumab OR Cisplatin OR Carboplatin OR Epirubicin OR Doxorubicin OR Cyclophosphamide OR fluorouracil)/exp |
| 11 | ('abi 007' OR 'abi007' OR 'abraxane' OR 'albumin bound paclitaxel' OR 'albumin-bound paclitaxel' OR 'anzatax' OR 'asotax' OR 'biotax' OR 'bms 181339' OR 'bms181339' OR 'bristaxol' OR 'britaxol' OR 'coroxane' OR 'formoxol' OR 'genexol' OR 'genexol pm' OR 'hunxol' OR 'ifaxol' OR 'infinnium' OR 'intaxel' OR 'mbt 0206' OR 'mbt0206' OR 'medixel' OR 'mitotax' OR 'nab paclitaxel' OR 'nanoparticle albumin bound paclitaxel' OR 'nsc 125973' OR 'nsc125973' OR 'oncogel' OR 'onxol' OR 'pacitaxel' OR 'paclitaxel nab' OR 'pacxel' OR 'padexol' OR 'parexel' OR 'paxceed' OR 'paxene' OR 'paxus' OR 'praxel' OR 'taxocris' OR 'taxol' OR 'taxus (drug)' OR 'taycovit' OR 'yewtaxan' OR 'paclitaxel'):ab,ti |
| 12 | ('daxotel' OR 'dexotel' OR 'docefrez' OR 'docetaxel accord' OR 'lit 976' OR 'lit976' OR 'n debenzoyl n tert butoxycarbonyl 10 deacetyltaxol' OR 'n tert butoxycarbonyl 10 deacetyl n debenzoyltaxol' OR 'nsc 628503' OR 'nsc628503' OR 'oncodocel' OR 'rp 56976' OR 'rp56976' OR 'taxoter' OR 'taxotere' OR 'texot' OR 'docetaxel'):ab,ti |
| 13 | ('5 deoxy 5 fluoro n4 (pentyloxycarbonyl) cytidine' OR 'apecitab' OR 'ecansya' OR 'ro 09 1978' OR 'ro 09-1978' OR 'ro 091978' OR 'ro09 1978' OR 'ro09-1978' OR 'ro091978' OR 'xeloda' OR 'capecitabine'):ab,ti |
| 14 | ('5 noranhydrovinblastine' OR 'anhydrovinblastine, 5 nor' OR 'anx 530' OR 'anx530' OR 'eunades' OR 'exelbine' OR 'kw 2307' OR 'kw2307' OR 'navelbin' OR 'navirel' OR 'vinbine' OR 'vinelbine' OR 'vinorelbine' OR 'vinorelbine ditartrate' OR 'vinorelbine tartrate'):ab,ti |
| 15 | ('e 7389' OR 'e7389' OR 'eribulin mesilate' OR 'eribulin mesylate' OR 'halaven' OR 'eribulin'):ab,ti |
| 16 | ('7, 11 dihydroxy 8, 8, 10, 12, 16 pentamethyl 3 [1 methyl 2 (2 methyl 4 thiazolyl) ethenyl] 17 oxa 4 azabicyclo [14.1.0] heptadecane 5, 9 dione' OR 'azaepothilone B' OR 'bms 247550' OR 'bms 247550 1' OR 'bms 247550-1' OR 'bms247550' OR 'bms247550 1' OR 'bms247550-1' OR 'ixempra' OR 'ixempra kit' OR 'nsc 710428' OR 'nsc710428' OR 'ixabepilone'):ab,ti |
| 17 | ('altuzan' OR 'avastin' OR 'nsc 704865' OR 'nsc704865' OR 'bevacizumab'):ab,ti |
| 18 | ('10 [(3 amino 2, 3, 6 trideoxy alpha levo arabino hexopyranosyl) oxy] 8 glycoloyl 7, 8, 9, 10 tetrahydro 6, 8, 11 trihydroxy 1 methoxy 5, 12 naphthacenedione':ab,ti OR '3 glycoloyl 1, 2, 3, 4, 6, 11 hexahydro 3, 5, 12 trihydroxy 10 methoxy 6, 11 dioxo 1 naphthacenyl 3 amino 2, 3, 6 trideoxy alpha levo arabino hexopyranoside':ab,ti OR '4 epiadriamycin':ab,ti OR '4 epidoxorubicin':ab,ti OR '4 epirubicin':ab,ti OR 'adriamycin, 4 epi':ab,ti OR 'binarin':ab,ti OR 'doxorubicin, 4 epi':ab,ti OR 'ellence':ab,ti OR 'epi-cell':ab,ti OR 'epiadriamycin':ab,ti OR 'epidoxo':ab,ti OR 'epidoxorubicin':ab,ti OR 'epidx':ab,ti OR 'epifil':ab,ti OR 'epilem':ab,ti OR 'epirubicin hydrochloride':ab,ti OR 'farmorrubicina rtu':ab,ti OR 'farmorubicin':ab,ti OR 'farmorubicin pfs':ab,ti OR 'farmorubicin rd':ab,ti OR 'farmorubicina':ab,ti OR 'farmorubicina cs':ab,ti OR 'farmorubicina r.d.':ab,ti OR 'farmorubicine':ab,ti OR 'imi 28':ab,ti OR 'nsc 256942':ab,ti OR 'pharmorubicin':ab,ti OR 'pharmorubicin pdf':ab,ti OR 'pharmorubicin pfs':ab,ti OR 'pharmorubicin r.d.f.':ab,ti OR 'pharmorubicin rds':ab,ti OR 'pharmorubicine':ab,ti OR 'pharmorubucin rd':ab,ti OR 'pidorubicin':ab,ti OR 'epirubicin'):ab,ti |
| 19 | ('14 hydroxydaunomycin' OR '14 hydroxydaunorubicin' OR 'a.d.mycin' OR 'adriablastin' OR 'adriablastina' OR 'adriablastina r.d.' OR 'adriablastine' OR 'adriacin' OR 'adriamicina' OR 'adriamicine' OR 'adriamycin' OR 'adriamycin hydrochloride' OR 'adriamycin p.f.s.' OR 'adriamycin pfs' OR 'adriamycin r.d.f.' OR 'adriamycin rd' OR 'adriamycin rdf' OR 'adriamycina' OR 'adriblastin' OR 'adriblastina' OR 'adriblastina cs' OR 'adriblastina pfs' OR 'adriblastine' OR 'adrim' OR 'adrimedac' OR 'adrubicin' OR 'amminac' OR 'caelix' OR 'caelyx' OR 'caelyx/doxil' OR 'carcinocin' OR 'dexorubicin' OR 'dox sl' OR 'doxil' OR 'doxil (liposomal)' OR 'doxolem' OR 'doxor lyo' OR 'doxorubicin hydrochloride' OR 'doxorubicin meiji' OR 'doxorubicin, liposomal' OR 'doxorubicine' OR 'doxorubin' OR 'evacet' OR 'farmiblastina' OR 'fi 106' OR 'fi106' OR 'ifadox' OR 'lipodox' OR 'liposomal doxorubicin' OR 'mcc 465' OR 'mcc465' OR 'myocet' OR 'nsc 123127' OR 'nsc123127' OR 'pegylated liposomal doxorubicin' OR 'polyethylene glycol-coated liposomal doxorubicin' OR 'rastocin' OR 'resmycin' OR 'rp 25253' OR 'rp25253' OR 'rubex' OR 'rubidox' OR 'sarcodoxome' OR 'tlc d 99' OR 'doxorubicin'):ab,ti |
| 20 | ('2 [bis (2 chloroethyl) amino] tetrahydro (2h) 1, 3, 2 oxazaphosphorine 2 oxide' OR '2 [bis (beta chlorethyl) amino] 1 oxa 3 aza 2 phosphacyclohexan 2 oxid' OR '2 [bis (2 chloroethyl) amino] 1 oxa 3 aza 2 phosphacyclohexane' OR '2 [bis (2 chloroethyl) amino] (2h) 1, 3, 2 oxazaphosphorinane 2 oxide' OR '2h 1, 3, 2 oxazaphosphorine 2 [bis (2 chloroethyl) amino] tetrahydro 2 oxide' OR 'alkyroxan' OR 'b 518' OR 'b 518 asta' OR 'b518' OR 'b518 asta' OR 'carloxan' OR 'ciclofosfamida' OR 'ciclolen' OR 'cicloxal' OR 'clafen' OR 'cyclo-cell' OR 'cycloblastin' OR 'cycloblastine' OR 'cyclofos amide' OR 'cyclofosfamid' OR 'cyclofosfamide' OR 'cyclophar' OR 'cyclophosphamid' OR 'cyclophosphamide isopac' OR 'cyclophosphamides' OR 'cyclophosphan' OR 'cyclophosphane' OR 'cyclostin' OR 'cyclostin n' OR 'cycloxan' OR 'cyphos' OR 'cytophosphan' OR 'cytophosphane' OR 'cytoxan' OR 'cytoxan lyophilized' OR 'endocyclo phosphate' OR 'endoxan' OR 'endoxan asta' OR 'endoxan-asta' OR 'endoxana' OR 'endoxon-asta' OR 'enduxan' OR 'genoxal' OR 'ledoxan' OR 'ledoxina' OR 'lyophilized cytoxan' OR 'mitoxan' OR 'n, n bis (2 chlorethyl) n o propylene phosphoric acid ester diamide' OR 'n, n bis (2 chloroethyl) n 3 (hydroxypropyl) phosphorodiamidic acid intramolecular ester' OR 'n, n bis (beta chlorethyl) n ortho trimethylenphosphorsaureesterdiamid' OR 'neosan' OR 'neosar' OR 'noristan' OR 'nsc 26271' OR 'nsc 2671' OR 'procytox' OR 'procytoxide' OR 'semdoxan' OR 'sendoxan' OR 'syklofosfamid' OR 'cyclophosphamide'):ab,ti |
| 21 | ('2, 4 dioxo 5 fluoropyrimidine' OR '5 fluoro 2, 4 pyrimidinedione' OR '5 fluoro uracil' OR '5 fluoropyrimidine 2, 4 dione' OR '5 fluorouracil' OR '5 fluoruracil' OR '5 fu' OR 'accusite' OR 'actino-hermal' OR 'adrucil' OR 'agicil' OR 'carac' OR 'cinkef u' OR 'effluderm' OR 'efudex' OR 'efudix' OR 'efurix' OR 'eurofluor' OR 'f6627' OR 'fivoflu' OR 'fluoro uracil' OR 'fluoroblastin' OR 'fluoroplex' OR 'fluorouracil 5' OR 'fluorouracil sodium' OR 'fluoruracil' OR 'fluouracil' OR 'fluoxan' OR 'flurablastin' OR 'fluracedyl' OR 'fluracil' OR 'fluracilium' OR 'fluril' OR 'fluro uracil' OR 'fluroblastin' OR 'fluroblastine' OR 'ifacil' OR 'nsc 18913' OR 'nsc 19893' OR 'nsc18913' OR 'nsc19893' OR 'oncofu' OR 'ribofluor' OR 'ro 2 9757' OR 'ro 2-9757' OR 'ro2 9757' OR 'ro2-9757' OR 'tolak' OR 'uflahex' OR 'utoral' OR 'verrumal' OR 'fluorouracil'):ab,ti |
| 22 | ('2 deoxy 2, 2 difluorocytidine' OR '2, 2 difluorodeoxycytidine' OR 'difluorodeoxycytidine' OR 'gemcitabine hydrochloride' OR 'gemcite' OR 'gemzar' OR 'ly 188011' OR 'ly188011' OR 'gemcitabine'):ab,ti |
| 23 | ('abiplatin' OR 'biocisplatinum' OR 'biocysplatinum' OR 'blastolem' OR 'briplatin' OR 'cddp ti' OR 'cis ddp' OR 'cis diamine dichloroplatinum' OR 'cis diaminechloroplatinum' OR 'cis diaminedichloroplatinum' OR 'cis diammine dichloroplatinum' OR 'cis diammine dichloroplatinum (ii)' OR 'cis diamminedichloroplatinum' OR 'cis diamminedichloroplatinum ii' OR 'cis dichloridiammineplatinum' OR 'cis dichloroadiamine platinum' OR 'cis dichlorodiamine platinum' OR 'cis dichlorodiamineplatinum (ii)' OR 'cis dichlorodiammine platinum ii' OR 'cis dichlorodiammineplatinum' OR 'cis dichlorodiammineplatinum (ii)' OR 'cis dichlorodiammineplatinum ii' OR 'cis dichlorodiammineplatinum (ii)' OR 'cis platinous diamino dichloride' OR 'cis platinum' OR 'cis platinum (ii) diamino dichloride' OR 'cis platinum (ii) diaminodichloride' OR 'cis platinum diamine dichloride' OR 'cis platinum diaminedichloride' OR 'cis platinum diamino dichloride' OR 'cis platinum diaminochloride' OR 'cis platinum diaminodichloride' OR 'cis platinum diaminodichloride ii' OR 'cis platinum diammine dichloride' OR 'cis platinum diamminedichloride' OR 'cis-platinum' OR 'cisplatin liposomal' OR 'cisplatin therapeutic implant' OR 'cisplatin-ebewe' OR 'cisplatine' OR 'cisplatino' OR 'cisplatinum' OR 'cisplatyl' OR 'citoplatino' OR 'cytoplatin' OR 'cytosplat' OR 'diamine dichloroplatinum' OR 'diaminodichloroplatinum' OR 'diamminedichloroplatinum' OR 'dichlorodiamine platinum' OR 'dichlorodiammineplatinum' OR 'docistin' OR 'elvecis' OR 'kemoplat' OR 'lederplatin' OR 'lipoplatin' OR 'liposomal cisplatin' OR 'mpi 5010' OR 'mpi5010' OR 'neoplatin' OR 'niyaplat' OR 'nk 801' OR 'noveldexis' OR 'nsc 119875' OR 'platamine' OR 'platamine rtu' OR 'platiblastin' OR 'platidiam' OR 'platimine' OR 'platinex' OR 'platinil' OR 'platinol' OR 'platinol aq' OR 'platinol-aq' OR 'platinoxan' OR 'platinum (11) diaminodichloride' OR 'platinum (ii) diaminodichloride' OR 'platinum (ii) diamino dichloride' OR 'platinum diamine dichloride' OR 'platinum diaminedichloride' OR 'platinum diaminodichloride' OR 'platinum diamminedichloride' OR 'platiran' OR 'platistil' OR 'platistin' OR 'platosin' OR 'randa' OR 'romcis' OR 'sicatem' OR 'spi 077' OR 'tecnoplatin' OR 'cisplatin'):ab,ti |
| 24 | ('1, 1 cyclobutanedicarboxylato) diammineplatinum' OR 'blastocarb' OR 'boplatex' OR 'carboplat' OR 'carboplatin a' OR 'carboplatin abic' OR 'carboplatin dbl' OR 'carboplatin lederle' OR 'carboplatino' OR 'carbosin' OR 'carbosin lundbeck' OR 'carbotec' OR 'carplan' OR 'CBDCA' OR 'cis diammine 1, 1 cyclobutanedicarboxylate platinum' OR 'cis diammine 1, 1 cyclobutanedicarboxylate platinum ii' OR 'cis diammine (1, 1 cyclobutanedicarboxylato) platinum' OR 'cis diammine (1, 1 cyclobutanedicarboxylato) platinum ii' OR 'cis diamminecyclobutane 1, 1 dicarboxylatoplatinum' OR 'cis (diammino) (1, 1 cyclobutanedicarboxylato) platinum' OR 'cycloplatin' OR 'delta west carboplatin' OR 'diammine cyclobutane 1, 1 dicarboxylatoplatinum' OR 'diamminecyclobutane 1, 1 dicarboxylatoplatinum' OR 'diamminecyclobutanedicarboxylatoplatinum' OR 'erbakar' OR 'ercar' OR 'ifacap' OR 'jm 8' OR 'jm-8' OR 'kemocarb' OR 'nsc 241240' OR 'oncocarbin' OR 'paraplatin' OR 'paraplatin rtu' OR 'paraplatin-aq' OR 'paraplatine' OR 'platinum cis diammine 1, 1 cyclobutanedicarboxylate' OR 'platinum cyclobutane 1, 1 dicarboxylatediammine' OR 'platinum diamino 1, 1 cyclobutanedicarboxylate' OR 'platinum diammine 1, 1 cyclobutanedicarboxylate' OR 'carboplatin'):ab,ti |
| 25 | #8 OR #9 OR #10 OR #11 OR #12 OR #13 OR #14 OR #15 OR #16 OR #17 OR #18 OR #19 OR #20 OR #21 OR #22 OR #23 OR #24 |
| 26 | #7 AND #25 |
| 27 | clinical trial'/exp OR 'clinical trial' |
| 28 | clinical trial (topic)'/exp OR 'clinical trial (topic)' |
| 29 | phase 3 clinical trial (topic)'/exp |
| 30 | phase 3 clinical trial'/exp |
| 31 | phase 2 clinical trial (topic)'/exp |
| 32 | phase 2 clinical trial'/exp |
| 33 | phase 1 clinical trial (topic)'/exp |
| 34 | phase 1 clinical trial'/exp |
| 35 | phase NEAR/4 (1 OR 2 OR 3 OR I OR II OR III) |
| 36 | #27 OR #28 OR #29 OR #30 OR #31 OR #32 OR #33 OR #34 OR #35 |
| 37 | #26 AND #36 |
| 38 | case report':ab,ti |
| 39 | clinical review':ab,ti |
| 40 | systematic review':ab,ti |
| 41 | [review]/lim |
| 42 | [letter]/lim |
| 43 | historical article':ab,ti |
| 44 | #38 OR #39 OR #40 OR #41 OR #42 OR #43 |
| 45 | #37 NOT #44 |
| 46 | **#45 AND [english]/lim AND [humans]/lim AND [1996-2016]/py** |
